# Secondary Metabolites of Actinomycetales as Potent Quorum Sensing Inhibitors Targeting Gram-Positive Pathogens: In Vitro and In Silico Study

**DOI:** 10.3390/metabo12030246

**Published:** 2022-03-15

**Authors:** Said E. Desouky, Mohammed Abu-Elghait, Eman A. Fayed, Samy Selim, Basit Yousuf, Yasuhiro Igarashi, Basel A. Abdel-Wahab, Amnah Mohammed Alsuhaibani, Kenji Sonomoto, Jiro Nakayama

**Affiliations:** 1 Department of Botany and Microbiology, Faculty of Science, Al-Azhar University, Cairo 11884, Egypt; dr_abuelghait@azhar.edu.eg; 2 Laboratory of Microbial Technology, Division of Systems Bioengineering, Department of Bioscience and Biotechnology, Faculty of Agriculture, Graduate School, Kyushu University, 744 Motooka, Nishi-ku, Fukuoka 819-0395, Japan; basucsmcri@gmail.com (B.Y.); sonomoto@kyudai.jp (K.S.); nakayama@agr.kyushu-u.ac.jp (J.N.); 3 Department of Pharmaceutical Organic Chemistry, Faculty of Pharmacy (Girls), Al-Azhar University, Cairo 11754, Egypt; alfayed_e@azhar.edu.eg; 4 Department of Clinical Laboratory Sciences, College of Applied Medical Sciences, Jouf University, Sakaka 72341, Saudi Arabia; sabdulsalam@ju.edu.sa; 5 Biotechnology Research Center, Department of Biotechnology, Toyama Prefectural University, 5180 Kurokawa, Imizu, Toyama 939-0398, Japan; yas@pu-toyama.ac.jp; 6 Department of Medical Pharmacology, College of Medicine, Assiut University, Assiut 7111, Egypt; basel_post@msn.com; 7 Department of Pharmacology, College of Pharmacy, Najran University, Najran 1988, Saudi Arabia; 8 Department of Physical Sport Science, College of Education, Princess Nourah bint Abdulrahman University, P.O. Box 84428, Riyadh 11671, Saudi Arabia; amalsuhaibani@pnu.edu.sa

**Keywords:** quorum sensing system, *agr* system, *fsr* system, *Enterococcus faecalis*, *Staphylococcus aureus*, actinomycetales metabolites, anti-virulence compounds, quorum sensing inhibitor, molecular docking

## Abstract

Anti-virulence agents are non-bacteriostatic and non-bactericidal emerging therapeutic options which hamper the production of virulence factors in pathogenic flora. In *Staphylococcus aureus* and *Enterococcus faecalis*, regulation of virulence genes’ expression occurs through the cyclic peptide-mediated accessory gene regulator (*agr*) and its ortholog *fsr* quorum sensing systems, respectively. In the present study, we screened a set of 54 actinomycetales secondary metabolites as novel anti-virulence compounds targeting quorum sensing system of the Gram-positive bacteria. The results indicated that four compounds, Phenalinolactones A–D, BU–4664LMe, 4,5-dehydrogeldamycin, and Questinomycin A, potentially inhibit the *agr* quorum sensing system and hemolytic activity of *S. aureus*. On the other hand, Decatromicin A and B, Okilactomycin, Rishirilide A, Abyssomicin I, and Rebeccamycin selectively blocked the *fsr* quorum sensing system and the gelatinase production in *E. faecalis* at sub-lethal concentrations. Interestingly, Synerazol uniquely showed the capability to inhibit both *fsr* and *agr* quorum sensing systems. Further, in silico molecular docking studies were performed which provided closer insights into the mode of action of these compounds and proposed that the inhibitory activity of these compounds could be attributed to their potential ability to bind to the ATP-active site of *S. aureus* AgrA. Taken together, our study highlights the potential of actinomycetales secondary metabolites with diverse structures as anti-virulence quorum sensing inhibitors.

## 1. Introduction

Quorum sensing (QS) is a dynamic process of cell-density-dependent regulation of gene expression that regulates various bacterial processes such as biofilm formation, virulence, and antibiotic resistance [1,2,3]. The accessory gene regulator (*Agr*) is a well-characterized central regulatory locus of the QS system in the Gram-positive bacteria which has been extensively studied in *S. aureus* [4,5]. On the other hand, the *fsr* QS system, an ortholog of the agr-like QS system, has been found in *E. faecalis* [6,7]. While the *agr* QS system is mediated by thiolactone-autoinducing peptides, the *fsr* QS system is mediated by gelatinase biosynthesis-activating peptide (GBAP) containing lactone [8]. *Staphylococcus aureus*, an opportunistic Gram-positive human pathogen, is the main cause of several infections, ranging from superficial skin and soft tissue infections to life-threatening toxic shock, pneumonia, endocarditis, and septicemia [9,10]. The *agr* locus mediates *S. aureus* infection by regulating the formation of biofilm and the release of different virulence factors [11,12]. In *S. aureus*, the *agr* locus consists of two divergent transcription units, RNAII and RNAIII, which are driven by the promoters *agr* P2 and *agr* P3, respectively [4]. RNAII encodes the AgrBDCA operon, the structural components of the QS system, whereas RNAIII encodes delta hemolysin, which functions as a regulatory of RNA that is involved in a series of virulence genes [11]. *Enterococci* are another group of opportunistic Gram-positive pathogens which reside in the gastrointestinal tract as commensals. They frequently cause several serious infections such as endocarditis, bacteremia, and urinary-tract infection [13,14]. The gelatinase production controlled by the *fsr* QS system has been reported to be a factor in the pathogenicity of enterococci [8]. The *fsr* locus consists of four genes, *fsrA*, *fsrB*, *fsrC*, and *fsrD*, which positively regulate the expression of gelatinase [15].

Methicillin-resistant *S. aureus* (MRSA) and vancomycin-resistant *Enterococci* (VRE) are among the most serious drug-resistant pathogens. Several antibiotics were shown to be ineffective against these pathogens, and therefore their therapeutic intervention become substantially tricky. In 2017, The World Health Organization (WHO) raised a strong concern for the development of novel antimicrobials against 12 multi-drug-resistant (MDR) pathogens, including MRSA and VRE as high-priority pathogens. The tremendous global spread of antibiotic resistance in pathogenic bacteria and the lack of potent and novel antibiotics against these pathogens can led to high morbidity and economic burden in the near future worldwide [16,17]. The QS system regulates the virulence of many drug-resistant opportunistic pathogens. Thus, the interference of QS has been considered as an alternative resort to the threats by these pathogens [18,19,20,21]. Indeed, targeting QS represents one of the emerging strategies for the development of anti-virulence agents which can attenuate virulence without a bactericidal effect [20,22,23,24]. Such QS-inhibitors could be used alone or synergistically in combination with antibiotics, which would reduce the emergency of wide-spectrum antibiotics and simultaneously minimize the risk for horizontal spread of drug-resistance genes [19,25,26,27,28]. Natural product-driven molecules comprise about 50% of the drugs recently used for clinical purposes [29]. Currently, several potent anti-QS agents have been identified which showed a promising therapeutic efficacy for impeding the pathogenicity of Gram-positive bacteria in vitro and in vivo levels, but they have not yet reached the clinical stage [30,31,32,33,34,35]. We have previously reported a novel set of QS-inhibitors which significantly attenuated *fsr* QS in *Enterococci* [30,36]. Furthermore, Desouky et al. have identified cyclodepsipeptides, obtained from actinomycetes culture extracts, as potent receptor antagonists for the *agr*/*fsr* QS systems [37]. Based on the above-mentioned facts and in continuation of our interest in identifying novel natural compounds with a unique mode of action, herein we extensively explore the inhibitory activity of a library of 54 natural antimicrobials from *Actinomycetes* spp. toward QS systems. Our study identified of a set of natural compounds which could potentially inhibit *agr/fsr* QS systems. Furthermore, we have performed a molecular docking study to investigate the binding affinity of the identified compounds towards the active site *of S. aureus* AgrA protein. This study represents a repertoire of natural compounds that are effective in controlling QS and advances knowledge towards the exploration of novel antimicrobial agents.

## 2. Results

### 2.1. Screening of Inhibitory Activity of Actinomycetales Metabolites against agr/fsr QS Systems

A set of 54 compounds of actinomycetales metabolites (Table S1) were screened for their activity to target the *agr*/*fsr* QS systems at 10 µg/mL using the *lux*/*gfp* dual reporter strain, *S. aureus* 8325-4 (pSB2035) and *E. faecalis* OG1RF (Table 1). Our initial screening results revealed that 14 compounds were able to potentially inhibit the *agr*/*fsr* QS systems. Interestingly, we found that some compounds possess selectivity to target the QS systems (Table 1). Phenalinolactones A–D (**35**), BU–4664LMe (**7**), Dehydrogeldamycin (**15**), and Questinomycin A (**50**) significantly inhibited the *agr* QS systems without influencing the growth of *S. aureus*. Phenalinolactones A–D and BU–4664LMe exhibited a potent inhibitory activity (80%), while Dehydrogeldamycin and Questinomycin A showed moderate activity (50% inhibition). On the other hand, Rebeccamycin (**44**), Rishirilide A (**45**), Abyssomicin I (**1**), Decatromicin A (**13**), Decatromicin B (**14**), and Okilactomycin (**33**) were shown to be selective and potent inhibitors for the *fsr* QS systems, with 80% inhibitory activity at the tested concentration without any considerable effect on the bacterial growth. Remarkably, Decatromicin A (**13**), Decatromicin B (**14**), and Okilactomycin (**33**) displayed considerable effects on the growth of *S. aureus* (80–50%) without affecting the *agr* QS system. Further, Rakicidin A (**45**), Rakicidin B (**9**), and Lysolipin (**28**) exhibited a substantial influence on the growth of both *S. aureus* and *E. faecalis* without any significant effect on the *agr*/*fsr* QS systems. Excitingly, BU–4664LMe (**7**) and Synerazol (**50**) demonstrated the ability to inhibit both the *agr* and *fsr* QS systems. While BU–4664LMe exhibited a substantial inhibitory activity towards the *fsr* QS system (80%), it also showed the ability to inhibit the *agr* QS system with 30% at the tested concentration. Conversely, Synerazol was shown to potentially inhibit the *agr* QS system and, in addition, it displayed a considerable inhibitory activity toward the *fsr* QS system. Based on these results, the hit compounds were divided into three different groups and were further investigated to confirm their activities.

### 2.2. Compounds Targeting Both agr and fsr Systems

**Figure 1:**
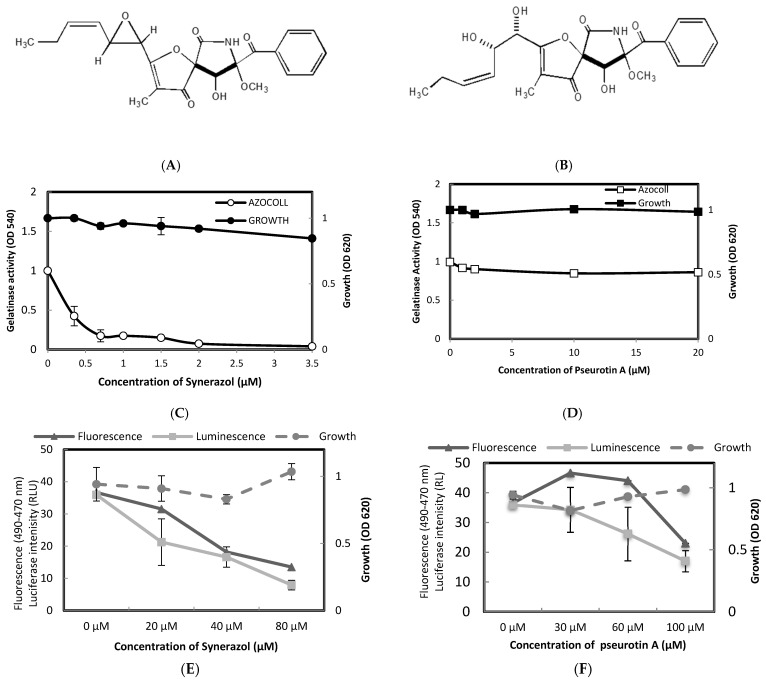
Compounds targeting *agr*/*fsr* QS systems. Structures of Synerazol (**A**) and Pseurotin A (**B**). (**C**,**D**) Effect of various concentrations of Synerazol and Pseurotin A on the cell growth and gelatinase production of *E. faecalis*. (**E**,**F**) Effect of Synerazol or Pseurotin A on *agr* expression in *S. aureus* 8325-4 (pSB2035).

Synerazol (**52**) is a known fungal metabolite that has been isolated from *Aspergillus fumigatus* (Figure 1A). To further investigate the dual activity of this compound on *agr* and *fsr* systems, we have examined the QS suppression activity of Pseurotin A (**53**) and Synerazol at different concentrations toward *E. faecalis* OG1RF and *S. aureus* 8325-4 (Figure 1B). The results revealed that Synerazol at 0.4 µM attenuated the gelatinase production of *E. faecalis* by 50% without inhibiting the bacterial growth (Figure 1C). Complete inhibition of gelatinase production was obtained at 2 µM of Synerazol. It is noteworthy that Azocoll was included in this experiment in order to avoid the possibility of direct inhibition of Synerazol on the gelatinase production. Additionally, Synerazol at 20 µM suppressed the expressions of GFP and luciferase in the dual reporter strain *S. aureus* 8325-4 (pSB2035) by 25% and 50%, respectively, without affecting bacterial growth (Figure 1E). On the other hand, Pseurotin A, which has been used as a negative control, showed no inhibition activity on both bacterial growth and gelatinase production of *E. faecalis* at concentrations up to 20 µM (Figure 1D). Further, Pseurotin A did not display a considerable inhibitory activity on the GFP expressions or luciferase in *S. aureus* at concentrations lower than 50 µM (Figure 1F). Taken together, these results indicate that Synerazol is a promising QS inhibitor which acts by targeting both *agr* and *fsr* QS systems. The structure of Synerazol and Pseurotin A is quite similar, whereby the former is the dehydrated form of Pseurotin A. This indicates that the Oxirane heterocycle is playing a critical role in the activity of the compound and that the Synerazol scaffold can be considered as a promising scaffold for the development of novel QS inhibitors.

### 2.3. Compounds Targeting fsr System

#### 2.3.1. Decatromicins A and B

**Figure 2:**
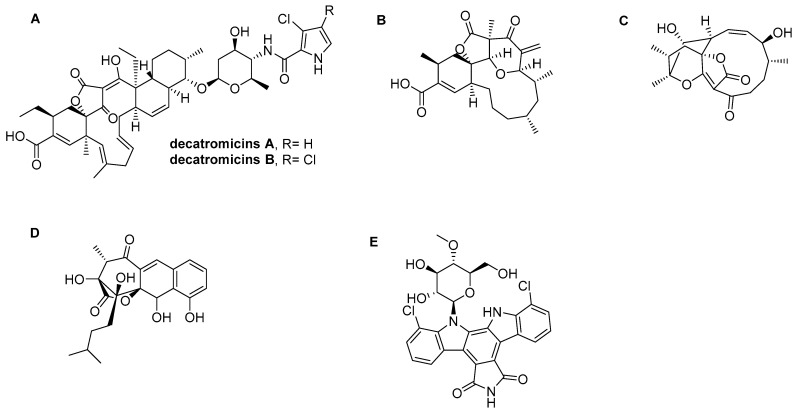
Chemical structures of compounds targeting the *fsr* QS system of *E. faecalis*. (**A**) Decatromicin A and B, (**B**) Okilactomycin, (**C**) Abyssomicin I, (**D**) Rishirilide A, (**E**) Rebeccamycin.

**Figure 3:**
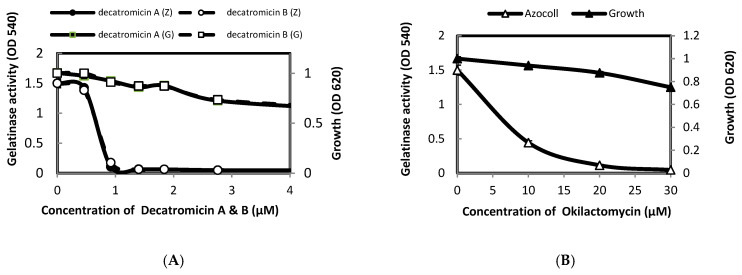

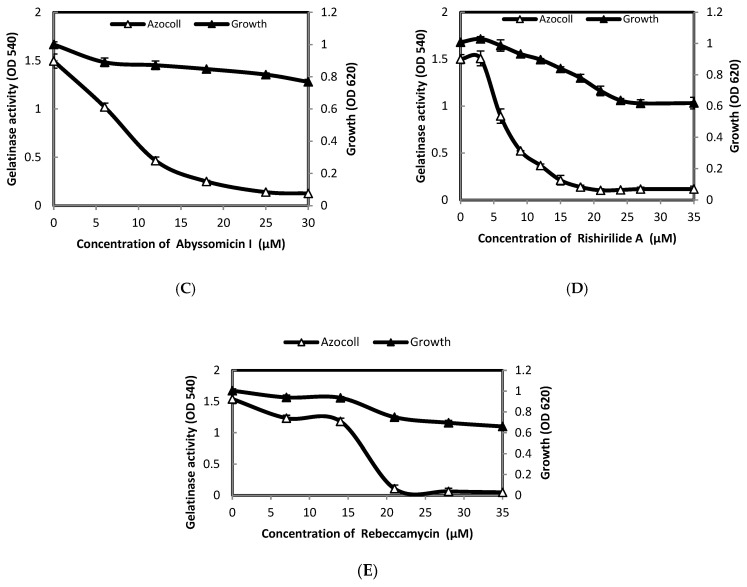
Effect of QS inhibitors on the gelatinase production and growth of *E. faecalis*. *E. faecalis* OG1RF was grown for 5 h in the absence and presence of QSIs at the indicated concentrations.

Decatromicins A and B (**13**, **14**) are antibiotics which were first isolated from *Actinomadura* MK73-NF4 by Momose et al. [38]. While the structures of decatromicins A and B are quite similar, consisting of macrocyclic lactone containing a tetronic acid, they differ in the pyrrole ring: the hydrogen atom in decatromicin A is replaced by a chlorine atom in decatromicin B (Figure 2A). Previous studies reported that decatromicins A and B have potent inhibitory activities toward the growth of *Staphylococci* with a MIC of 3.13 μM [38]. In our study, these compounds demonstrated a sub-micromolar inhibitory activity toward the gelatinase production of *E. faecalis* with a minor effect on the bacterial growth (Figure 3A). Full inhibition of gelatinase production was achieved at 1 μM of compound. These results indicated that decatromicins A and B antibiotics could be considered as influential drugs against the *fsr* QS system.

#### 2.3.2. Okilactomycin

Okilactomycin (**33**) is a well-known antibiotic against Gram-positive bacteria which is isolated from *Streptomyces griseoflavus* [39] and *Streptomyces scabrisporus* [40]. Structurally, it has a cyclohexene ring with a spirocenter and a 2,6-*cis*-tetrahydropyranone moiety (Figure 2B). Okilactomycin potentially inhibited the growth of *E. faecalis* IID 682 and *S. aureus* FDA209PJC-1 with a MIC of 250 and 125 μM, respectively [40]. In our screening, Okilactomycin displayed a significant inhibitory activity toward the *fsr* QS system. Toward this end, we further investigated the effect of Okilactomycin on the gelatinase production and the bacterial growth. As shown in Figure 3B, Okilactomycin inhibited 70% of the gelatinase production of *E*. *faecalis* at a 10 μM concentration without a dramatic influence on the bacterial growth. These results revealed that the mode of action of Okilactomycin may involve targeting the bacterial *fsr* QS system.

#### 2.3.3. Abyssomicin I

Abyssomicin I (**1**) is a newly modified polycyclic polyketide (Figure 2C), which was isolated from *Streptomyces* sp. CHI39 [41]. Although it has been reported that Abyssomicin I inhibits the growth of *S. aureus* with a MIC of 50 µM, we did not observe a substantial effect on the growth of *E. faecalis* at concentrations up to 30 µM. On the other hand, Abyssomicin I exhibited a potent inhibitory activity against the gelatinase production regulated by the *fsr* QS system with a MIC of 12 µM without affecting the bacterial growth (Figure 3C).

#### 2.3.4. Rishirilide A

Rishirilide A (**45**) is a plasmin antagonist with a highly oxygenated anthracene skeleton which is produced by *Streptomyces rishiriensis* OFR-1056 (Figure 2D) [42]. As depicted in Figure 3D, Rishirilide A did not display a significant growth inhibitory activity against *E. faecalis* up to a 35 µM concentration. On the other hand, Rishirilide A drastically inhibited the gelatinase production of *E. faecalis* at an 8 µM concentration (70% inhibition). As for the above inhibitors, Rishirilide A did not display a direct inhibitory activity towards the gelatinase of *E. faecalis*.

#### 2.3.5. Rebeccamycin

Rebeccamycin (**44**) is an antibiotic which is produced by *Saccharothrix aerocolonigenes* (Figure 2E) (**55**). It has a wide range of biological activity, ranging from antitumor to antimicrobial activity [17]. Rebeccamycin displayed a considerable inhibitory activity toward the growth of *S*. *aureus* A953 with a MIC of 2 µM (55). In our investigations, Rebeccamycin did not show a significant effect on the growth of *E. faecalis* at the concentrations up to 20 µM (Figure 3E). However, it slightly attenuated the gelatinase production (up to 27%) at 15 µM without affecting cell growth (Figure 3E). Further, a direct inhibitory activity toward the gelatinase of *E. faecalis* was not observed. Interestingly, Rebeccamycin completely inhibited the gelatinase production of *E. faecalis* at 20 µM, however this was accompanied by a reduction of bacterial growth (20% inhibition). 

### 2.4. Compounds Targeting agr System

#### 2.4.1. Phenalinolactones A–D

**Figure 4:**
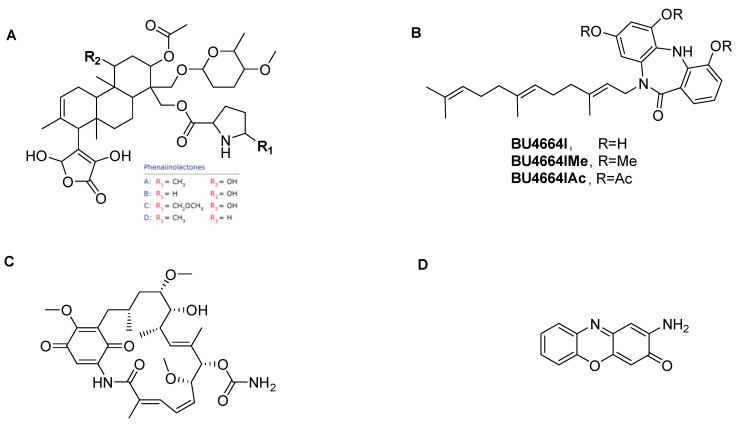
Chemical structures of compounds targeting the *agr* QS system of *S. aureus*. (**A**) Phenalinolactones A–D, (**B**) BU–4664L, (**C**) Geldanamycin, (**D**) Questinomycin A.

**Figure 5:**
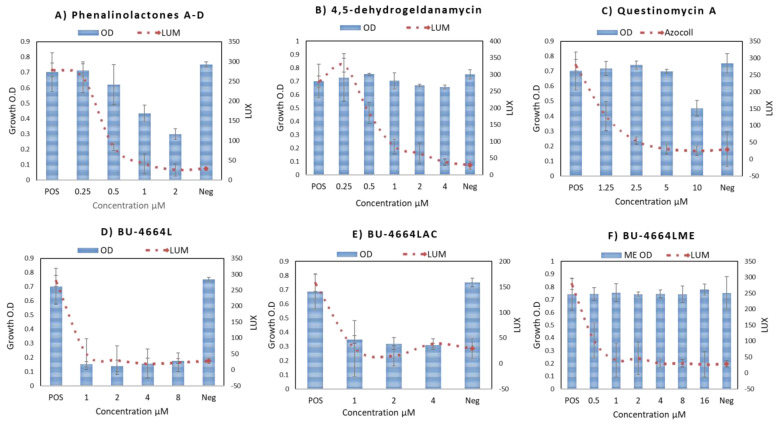
Effect of Phenalinolactones A–D, 4-5-dehydrogeldnamycin, Questinomycin A, BU–4664L, BU–4664LAc, and BU–4664LMe on *agr* expression and cell growth in *S. aureus* 8325-4 (pSB2035).

Phenalinolactone is an antibiotic produced by *Streptomyces* sp. U4664l and displays a potent inhibitory activity toward tumor, invasion, and angiogenesis [43]. This compound significantly inhibited the *agr* system in *S. aureus* and affecting the cell growth (Figure 4A and Figure 5A).

#### 2.4.2. 4,5-Dehydrogeldanamycin

Geldanamycin (**15**) was first isolated from *Streptomyces hygroscopicus* in 1970 [44]. This compound plays an important role in attenuating the highly virulent H5N1 influenza virus infection by reducing the host’s inflammatory responses [45]. Our result showed that 4,5-dehydrogeldanamycin (derivative of Geldanamycin) reduced the expression of the *agr* QS system in *S. aureus* 8325-4 (pSB2035) at the concentration of 2.5 µg/mL by reducing luciferase production by 93.3% without affecting cell growth (Figure 4C). This compound attenuated the anti-hemolytic activity of the *S. aureus* ATCC 29213 strain at the concentration of 5 µg/mL (Figure 5B).

#### 2.4.3. Questinomycin A

Questiomycin A (**40**) has been reported as a causative agent of apoptotic cell death in the gastric and colon cancer cell lines (Figure 4D) [46]. Our results showed that Questinomycin A inhibited the regulation of *agr* QS by 87.5%, as indicated by luciferase induction of *S. aureus* 8325-4 (pSB2035) at a 5 µg/mL concentration without affecting cell growth. When the activity of this compound was assessed against *S. aureus* ATCC 29213, it exhibited a reduction of relative hemolysis of this strain of 21.2% (Figure 5C).

#### 2.4.4. BU–4664L, Me, Ac

BU–4664L (**5**) is a secondary metabolite initially discovered from the actinomycete of the genus *Micromonospora* (Figure 4B) [47]. This compound is described as a heterocyclic core, dibenzodiazepinone, modified with a farnesyl carbon chain (Figure 5D). BU–4664L is comprised of two distinct substructures, the dibenzodiazepine core and the aliphatic farnesyl sidechain. In order to assess which part is responsible for bioactivities, two derivatives of BU–4664L were obtained [48], BU–4664LAc and BU–4664LMe by acetylation and methylation of BU–4664L, respectively (Figure 4B). We analyzed the QSI activity of BU–4664L against the *agr* QS system of *S. aureus* 8325-4 (pSB2035) at concentrations ranging from 1 to 8 µM. It was observed that BU–4664LMe inhibited luciferase up to 90% without affecting cell growth, whereas BU–4664L and BU–4664LAc affected the cell growth at a concentration of 5 µg/mL for both (Figure 5D–F). 

### 2.5. Antihemolytic Activity

**Figure 6:**
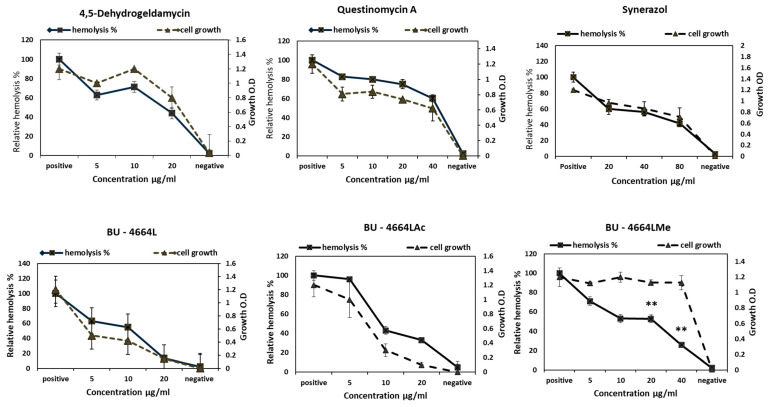
Anti-hemolytic activity of 4,5-dehydrogeldnamycin, Questiomycin A, Synerazol, BU–4664L, BU–4664LMe, and BU–4664LAc on *S. aureus* ATCC 29213. Significant differences are indicated by ** *p* < 0.05.

The tested compounds exhibited diverse effects against the hemolytic activity of *S. aureus* ATCC 29213 (Figure 6). BU–4664LMe was the most potent sample tested. It significantly (*p* < 0.05) reduced the hemolytic activity of *S. aureus* by up to 74.4% and 47.3% at concentrations of 40 and 20 µg/mL, respectively, without combating the bacterial cell growth in a dose-dependent manner. On the other hand, other tested compounds were neglected either for their effect on the bacterial growth at the same concentrations or for exhibiting non-significant hemolytic activities against *S. aureus* (Figure 6).

### 2.6. In Silico Molecular Modeling Study

**Figure 7:**
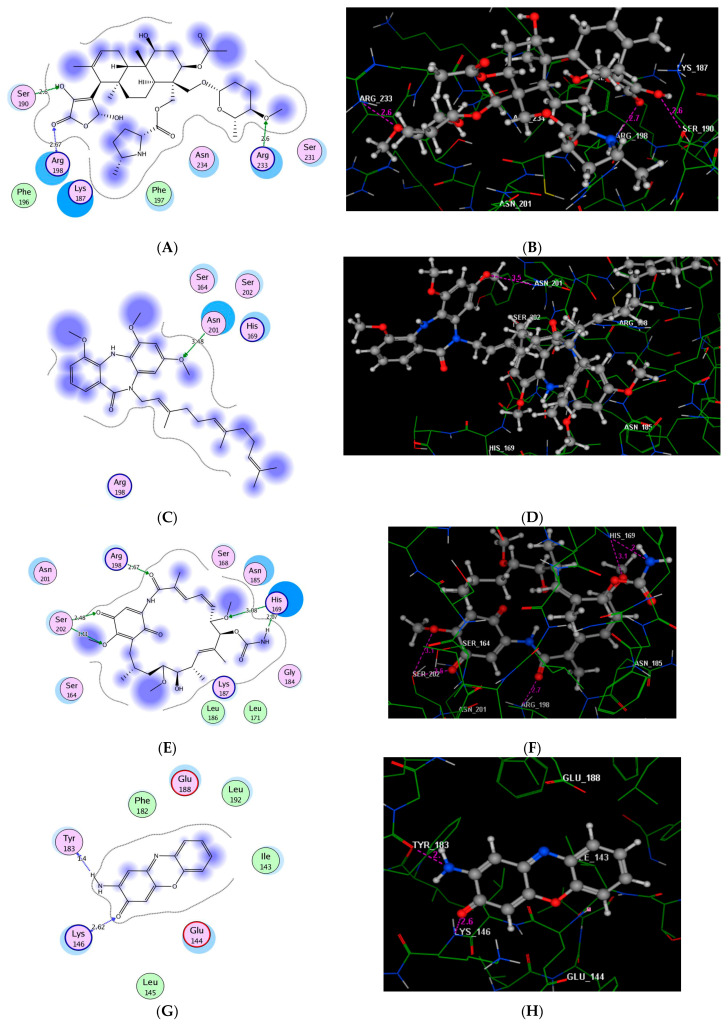

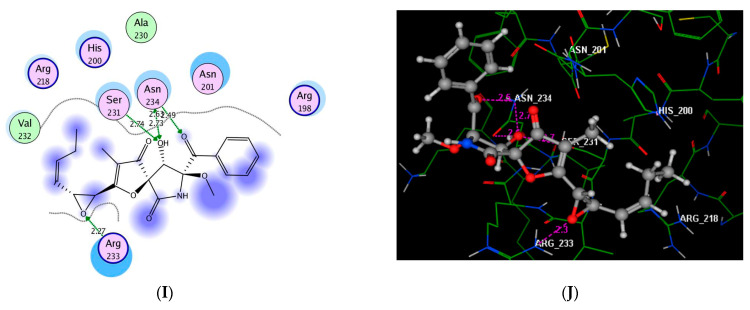
Illustration of the 2D (**A**) and 3D (**B**) binding patterns of Phenalinolactones A–D (**A**,**B**), BU–4664LMe (**C**,**D**), 4,5-dehydrogeldamycin (**E**,**F**), Questinomycin A (**G**,**H**), and Synerazol (**I**,**J**), into the ATP-active pocket of *AgrA* (PDB code: *3BS1*). The hydrogen bonds are illustrated as dotted pink arrows (C atoms are colored green, S yellow and O red).

To further explore the inhibitory potency of the discovered potent inhibitors (Phenalinolactones A–D (**35**), BU–4664LMe (**7**), 4,5-dehydrogeldamycin (**15**), Questinomycin A (**40**), and Synerazol (**50**)), a detailed in silico molecular docking simulation study was performed toward the active site of *Staphylococcus aureus AgrA* targets (PDB code: *3BS1*) [49,50]. The investigation of the *AgrA* binding site revealed the key amino acid residues that interact with the DNA backbone (Ser168, Arg170, Tyr183, Lys187, Ser202, Arg218, and Asn234) together with the three amino acid residues that perform direct, base-specific contacts (His169, Asn201, and Arg233) [50]. All tested compounds (Phenalinolactones A–D, BU–4664LMe, 4,5-dehydrogeldamycin, Questinomycin A, and Synerazol) showed a high binding affinity to the ATP-active site of *AgrA*, with energy scores of −10.43, −10.16, 10.20, −8.55, and −11.10 kcal/mol, respectively. As shown in Figure 6, Phenalinolactones A–D formed a stable binding mode to the active site of the ATP pocket by forming a hydrogen bond between the oxygen of methoxy group at the pyran ring and the Arg233 residue (distance: 2.60 Å). Further, the 2,4-dihyroxy-5-oxofuran contributed to the binding fixation through two H-bonds, one between the carbonyl oxygen and the Arg198 residue and the other between the hydroxyl group and the Ser190 residue (distance: 2.67 and 2.60 Å, respectively). As show in Figure 7C,D, the methoxy group at the dibenzodiazepinone scaffold maintained the improved potency in BU–4664LMe through hydrogen bond formation between the oxygen atom and the sidechain of the important Asn201 residue (distance: 3.48 Å). Additionally, the centroid of the 4,6-dimethoxybenzene ring was facing the essential amino acids Ser164, His169, and Ser202. 

The adjacent methoxy and carbamate fragments play an essential role in the fixation of 4,5-dehydrogeldamycin within *AgrA* through the formation of two H-bonds with the sidechain of His169: one is an acceptor with the oxygen of methoxy and the other is a donor with the proton of NH_2_ group (distance: 3.08 and 2.07 Å, respectively). Moreover, the cyclohexadiene assisted the binding via two hydrogen bond acceptors between the oxygens of carbonyl and methoxy groups with the sidechain of Ser202 (distance: 2.48 and 3.11 Å, respectively). Additionally, the oxygen of the amide moiety exhibited an H-bond acceptor with the sidechain of Arg198 (distance: 2.67 Å) (Figure 7E,F). The phenoxazine scaffold of Questinomycin A demonstrated the ability to embed and bind to the ATP-active pocket of *AgrA* through the formation of two main hydrogen bonds. As illustrated in Figure 6, the NH_2_ group demonstrated an H-bond donor to the Tyr183 residue (distance: 1.40 Å). On the other hand, the carbonyl oxygen in the scaffold bonded as an H-bond acceptor to the Lys146 residue in the active site (distance: 2.62 Å) (Figure 7G,H). 

The molecular modeling of Synerazol revealed a high binding affinity toward the ATP-active pocket of *AgrA*, which was demonstrated by the ability to form several H-bonds to the active site of the protein. In this regard, the oxygen atom of the oxirane moiety demonstrated an H-bond acceptor with the Arg233 residue (distance: 2.27 Å). The binding mode was further stabilized by several other hydrogen bonds afforded by the Asn234 and Ser231 residues. While the Asn234 residue served as an H-bond donor with the two oxygens of benzoyl and hydroxyl fragments (distance: 2.49 and 2.73 Å, respectively), it also acted as an H-bond acceptor with the hydroxyl oxygen (distance: 2.63 Å). On the other hand, the Ser231 residue demonstrated an H-bond donor site with the hydroxyl oxygen (distance: 2.74 Å) (Figure 7I,J).

## 3. Discussion

Several antipathogenic compounds targeting QS in Gram-negative bacteria have been discovered [20,51]. However, there are limited QSIs against Gram-positive bacteria reported in the literature. Previously, our research group discovered ambuic acid, a known antifungal compound which inhibited the biosynthesis of cyclic peptide autoinducers of *S. aureus* and *Listeria innocua* as well as GBAP of *E. faecalis* [52]. We have also identified actinomycete metabolites such as alasso peptide, siamycin, and cyclodepsi peptides of WS9326s as potent QSIs. Siamycin was found to inhibit the *fsr* QS of *E. faecalis*, whereas WS9326s was found to have anti-virulence activity by obstructing the *agr*, *fsr*, and VirSR QS of *S. aureus*, *E. faecalis*, and *Clostridium perfringens*, respectively [37]. These findings suggested that ambuic acid and WS9326s offer wide-spectrum QSIs for Gram-positive pathogens. The research has motivated us to explore more secondary metabolites which can effectively attenuate *agr* and *fsr* QS systems of *S. aureus* and *E. faecalis*, respectively. Indeed, the three-step high-throughput screening system exhibited in this study will accelerate the discovery of a wide range of drug candidates, and may offer a new type of antimicrobial chemotherapy and also knowledge leading to the rational design of QSIs in a next step. In this study, a total of 10 compounds were discovered as new QSIs targeting *agr* or *fsr* QS systems (Table 1). The QSI Phenalinolactone, produced by *Streptomyces* sp. Tü6071, has been previously reported to have antibacterial activity [53]. Questiomycin A, an analog of actinomycin D, is known to have anticancer properties and cause apoptosis in the different cancer cell lines, but the mechanism of its action is unknown [54]. We observed in this study that Phenalinolactones A–D and Questiomycin A inhibit the *agr*-type QS systems and blood hemolysis in *S. aureus*.

Rebeccamycin is known as a topoisomerase I inhibitor, which has been previously isolated from *Saccharothrix aerocolonigenes*, Gram-positive bacteria (55). It has been shown to have strong antitumor activity against mouse B16 melanoma cells (IC_50_ = 500 nM) and P388 leukemia cells (IC_50_ = 480 nM), and it also has antibacterial properties [55]. Rebeccamycin and its analogues are currently undergoing clinical phase II trials for the treatment of lung cancer, liver cancer, breast cancer, lymphoma, retinoblastoma, kidney cancer, and ovarian cancer. This study showed significant inhibition of *fsr* QS in *E. faecalis* without influencing the *agr* system and bacterial growth of *S. aureus*.

Decatromicins A and B, initially isolated from *Actinomadura* sp. MK73-NF4, have been reported to inhibit the growth of MRSA and other Gram-positive bacteria [38]. These compounds strongly inhibited the *fsr*-induced gelatinase production with a neglected shift in the bacterial growth. The difference between Decatromicins A and B is the substitution of hydrogen by a chlorine atom attached to the pyrrole ring (Figure 2A). We observed that there is no difference in effective dose between Decatromicins A and B for growth inhibition or QSI activity against *E. faecalis* OG1RF. Our results indicated that the chlorine atom or halogenation are not at all important for the QSI mechanism of Decatromicins. Okilactomycin, a polyketide antibiotic produced by *Streptomycesgriseoflavus*, is known to have antitumor activity and weak antimicrobial activity against Gram-positive bacteria. This study indicated that Okilactomycin strongly inhibited GelE production of *E. faecalis* OG1RF at a 20 µM concentration, with a slight attenuation in the bacterial growth. Previously, Zhang et al. investigated that Okilactomycin targets small ribosomal protein S4 in the growth inhibition mechanism of *S. aureus* [40]. 

Notably, Synerazol efficiently inhibited both *fsr* and *agr* QS systems. The structure of Synerazol was interesting in terms of its structure and activity relationship as its analogue, Pseurotin A, did not show any QSI activity (Figure 1D). The structural difference between these two compounds is only diol in Pseurotin A instead of epoxide of Synerazol at the same position (Figure 1A,B), suggesting that the epoxide is associated with the inhibitory activity against the cyclic peptide-mediated QSI in Gram-positive bacteria. From the previous results, it was found that most of the examined compounds exhibited moderate to high activities. Synerazol showed a unique activity as a global regulator of both *fsr* and *agr* QS systems. The higher inhibitory activity of Synerazol than Pseurotin A could be attributed to the presence of the Oxirane fragment, which is important in the binding with Arg233. With the opening of the Oxirane ring in Pseurotin A (which is typical in the structure of Synerazol, except for the Oxirane ring), the activity is totally diminished. The most active compounds targeting *agr* are Phenalinolactones A–D, BU–4664L, Geldnamycin, and Questinomycin A.

In view of the structural composition of the compounds, we found that most of them contain the methoxy group, which is of great importance in the process of linking with the amino acids. For example, Phenalinolactones can form a hydrogen bond acceptor between the oxygen of the methoxy group at the pyran ring and the sidechain of Arg233. Furthermore, among the three derivatives of BU–4664L (R = H, COCH_3_, CH_3_), it was noted that BU–4664LMe bearing three methoxy groups at the dibenzodiazepinone scaffold revealed an excellent inhibitory activity due to the presence of the three methoxy groups, which have a +M effect rather than OH and COCH_3_ which have a–I effect. Moreover, Geldnamycin showed binding of methoxy and carbamate fragments within *AgrA* through the formation of two H-bonds with the sidechain of His169. The only different structure was Questinomycin A, which lacks the presence of a methoxy group, and here, the binding occurred through the carbonyl oxygen with amino acid Lys146, while the amino group revealed an H-bond donor with the backbone of Tyr183. On the other hand, we found some other compounds targeting *fsr*, which are Decatromicins A and B, Okilactomycin, Abyssomicin I, Rishirilide A, and Rebeccamycin. Four of them, Decatromicins A and B, Okilactomycin, Abyssomicin I, and Rishirilide, have a furanone moiety in addition to other functional groups, which may be important in activity, as the furanone (**21**) itself is inactive. The important groups are the carboxylic group, which is present in Decatromicin (A and B) and Okilactomycin, and also the sugar moiety, that is present in Decatromicins A and B. Rebeccamycin, which contains a sugar moiety in addition to pyrrole rings, which is the bioisostere for the furan ring, also represents higher activity.

## 4. Materials and Methods

### 4.1. Bacterial Starins and Growth Conditions

*E. faecalis* OG1RF was used as an isogenic gelatinase-positive strain to assay the QSI activity of samples for screening [56]. *E. faecalis* OG1RF was cultured in 36.4 g/L of Todd–Hewitt broth (THB) (Oxoid, Basingstoke, Hampshire, United Kingdom) at 37 °C with gentle agitation. *S. aureus* 8325-4 (pSB2035) [18] and *S. aureus* 12600^T^ [19] were cultured in Luria–Bertani (LB) broth (10 g of tryptone, 5 g of yeast extract, and 10 g of NaCl per liter) at 37 °C with gentle agitation. For *S. aureus* 8325-4 (pSB2035), 7 µg/mL of chloramphenicol for plasmid selection was added to the medium. *S. aureus* 8325-4 (pSB2035) was procured from Prof. Paul Williams at The University of Nottingham, UK. *S. aureus* ATCC 29213, a highly hemolysin-producing strain [12], was used for the antihemolytic assay.

### 4.2. Compound Library

Fifty-four compounds of different chemical structures, isolated from *Actinomycetales* sp., were obtained from the Biotechnology Research Center at Toyama Prefectural University [41]. These compounds were investigated for anti-*fsr* and anti-*agr* activities.

### 4.3. Screening and Assessment of E. faecalis fsr System Inhibition

The assay for the inhibition of the *E. faecalis* *fsr* QS system was carried out essentially as described previously [57]. For the overnight grown culture of *E. faecalis* OG1RF (3 µL), 0.5 McFarland (1.5 × 10^8^ CFU) was inoculated into 0.5 mL of THB medium containing 5 μg of the tested compounds (54 compounds) dissolved in methanol and was cultured for 5 h at 37 °C with gentle shaking. The growth was measured at an OD of 600 nm (OD_600_). After centrifugation at 9100× *g* for 5 min, 40 µL of culture supernatant was collected and subjected to the gelatinase assay using azocoll (Calbiochem, San Diego, CA, USA) as a substrate, as described previously [30,36,41]. Briefly, 40 µL of *E. faecalis* culture supernatant was added to 0.8 mL of azocoll suspension, incubated for 4 h with constant mixing (170 rpm), and centrifuged at 20,000× *g* for 5 min, and the OD of the supernatant was then measured at OD_540_. The inhibitory effect was assessed by evaluating the growth of the culture with tested compounds and the non-cultured control sample, which was considered as a negative control. Inhibition of growth by more than 50% was judged as significant inhibition and the compound was considered for further studies. The experiment was performed in triplicate.

### 4.4. Screening and Assessment of S. aureus agr System Inhibition

The assay for the inhibition of the *S. aureus agr* QS system was carried out using the *S. aureus agr* reporter strain, 8325-4, which carries plasmid pSB2035 encoding luciferase and GFP genes under the agr P3 promoter. For the inhibitory assay of the *agr* system, in an overnight culture of *S. aureus* 8325-4 (pSB2035), 0.5 McFarland (1.5 × 10^8^ CFU) was diluted 1 to 50 into 200 µL of fresh LB broth containing 2 μg of the tested compounds (54 compounds) and then cultured in a 96-well titer plate with shaking at 120 rpm. For the positive and negative controls, *S. aureus* 8325-4 (pSB2035) and *S. aureus* 12600 were cultured in the same way without compounds, respectively. After 7 h, the OD of the culture at 620 nm was measured by a microtiter plate reader (Immuno Mini NJ-2300; Nihon InterMed, Tokyo, Japan). Simultaneously, bioluminescence of the culture was quantified by a luminescence image analyzer (LAS-4000mini; Fuji Photo Film, Tokyo, Japan) with FUJIFILM Multi-Gauge software. If the OD_620_ was less than 50% of the positive control, the sample was judged to have growth inhibitory activity and removed from the batch for the QSI assay. The induction level of luciferase was calculated by subtracting the luminescence of the negative control. The full induction level was calculated by subtracting the luminescence of the negative control from that of the positive control. An inhibitory effect of more than 90% was judged as significant inhibition. To quantify the GFP expression level, the culture was collected from the well and the cells were harvested by centrifugation at 13,000× *g* for 2 min, washed twice in an equal volume of phosphate-buffered saline (PBS), and then suspended into 600 µL of PBS. The fluorescence (excitation at 470 nm (F_470_) and 490 nm (F_490_) and emission at 525 nm) of each sample was measured by a fluorescence spectrophotometer (F-7000; Hitachi High technologies). The fluorescence of GFP was represented by F_490_–F_470_. The induction level of GFP was calculated by subtracting the F_490_–F_470_ value of the negative control. The full induction level was calculated by subtracting the F_490_–F_470_ value of the negative control from that of the positive control. The experiment was performed separately, three times.

### 4.5. Quantitative Assessment of Anti-Hemolytic Effect

Hemolysin production was quantitatively assessed according to the previous method with some modifications [37]. The overnight culture of *S. aureus* ATCC 29213 was diluted to 1:100 in TSB and then grown with or without sublethal concentrations of the investigated compounds at 37 °C for 16 h with shaking at 250 rpm. Then, 100 μL of the cell culture was added into the diluted human red blood cells that had previously been separated by centrifugation at 900× *g* for 5 min, washed with PBS buffer three times, and then diluted to 3% in PBS buffer. For hemolytic activity, the mixture was incubated at 37 °C for 3 h with 250 rpm shaking. The supernatant was collected by centrifugation at 16,600× *g* for 10 min and the optical density (OD) was measured at 543 nm [41]. The anti-hemolysin activity of the tested compounds was determined by comparing the hemolytic activity of *S. aureus* cultured with/without the investigated compounds and then calculating the ratio [42]. The experiment was performed in triplicate.

### 4.6. Molecular Docking Study

The molecular docking simulation study was performed using Molecular Operating Environment (MOE^®^) 2008.10 software [58,59,60,61,62,63]. The crystal structure of *Staphylococcus aureus* AgrA (PDB code: 3BS1) was retrieved from Protein Data Bank [49]. The 2D structures of the five potent compounds: phenalinolactones A–D, BU–4664LMe, 4,5-dehydrogeldamycin, questiomycin A, and synerazol, were drawn by ChemDraw. Then, the protonated 3D structures were built using standard bond angles and lengths, with the MOE 10.2008 software. Geometry optimization and energy minimization were performed to employ the Conf Search module in MOE, and then the MOE files were saved to be available for the docking process. The structure of AgrA was prepared for molecular docking using the Protonate 3D protocol in MOE with the default options. The Triangle Matcher placement method and London dG scoring function were applied in the docking protocol according to the reported procedure [58,59,60,64,65,66,67].

### 4.7. Statistical Analysis

Three replicates were performed for each assay and all resulted values were the averages of three independent experiments. To analyze the differences between a sample and the corresponding control, Student’s t-test was used. Differences were considered significant if the *p*-values were <0.05.

## 5. Conclusions

This study highlighted the antimicrobial compounds with potential anti-QS activity which will subsequently pave the way to cope with bacterial infections. This study has gained substantial knowledge about the chemical structures of QSIs capable of attenuating the virulence of potential drug-resistant Gram-positive bacteria. In this context, resources of microbial secondary metabolites have a high potential as the screening library for anti-virulence agents targeting *agr* and *fsr* QS of Gram-positive bacteria. Accordingly, a set of compounds have been discovered which showed the ability to potentially target the *agr* and/or *fsr* QS systems of the bacteria. Further studies should be performed in the future to investigate the potency of the discovered compounds on sensitive and resistant strains.

## Figures and Tables

**Table 1:** Compound library screening against luciferase activity and gelatinase production.

No.	Compound	*S. aureus* 8325-4		*E. faecalis* OG1RF	
		Growth	Luciferase	Growth	Gelatinase
1	Abyssomicin I	-	-	-	+++
2	Bb47-6-4	-	-	-	-
3	Bg32-12c	-	-	-	-
4	Borrelidin	-	-	-	-
5	Bu–4664L	-	-	-	-
6	BU–4664LAc	-	-	-	-
7	BU–4664LMe	-	+++	-	+
8	Chi15a	-	-	-	-
9	Chi93a	-	-	-	-
10	Chromone	-	-	-	-
11	Collismycin	-	-	-	-
12	Coumarine	-	-	-	-
13	Decatromicin A	+++	NA	-	+++
14	Decatromicin B	+++	NA	-	+++
15	Dehydrogeldamycin	-	++	-	-
16	Derivative EX1	-	-	-	-
17	Derivative EX2	-	-	-	-
18	Enterocin	-	-	-	-
19	Mycolic acid	-	-	-	-
20	Fistupyrone	-	-	-	-
21	Furanone	-	-	-	-
22	Gmku	-	-	-	-
23	HF599 Maleimide	-	-	-	-
24	Isi1-1-C	-	-	-	-
25	Leptomycin A	-	-	-	-
26	Lupinacidin C	-	-	-	-
27	Lydicamycin	-	-	-	-
28	I4,5-Lysolipin	+++	NA	+++	NA
29	Maklamicin	+++	NA	-	-
30	Mucidone	-	-	-	-
31	Myxochelinamide	-	-	-	-
32	Nocardimicin H	-	-	-	-
33	Okilactomycin	+	NA	-	+++
34	Pseurotin A	-	-	-	-
35	Phenalinolactones A–D	-	+++	-	-
36	Pradimicin S	-	-	-	-
37	Preussin	-	-	-	-
38	Pristinamycin IIA	-	-	-	-
39	Pyridoxatin	-	-	-	-
40	Questinomycin A	-	++	-	-
41	Radiciccol	-		-	-
42	Rakicidin A	++	NA	+++	NA
43	Rakicidin B	++	NA	+++	NA
44	Rebeccamycin	-	±	-	+++
45	Rishirilide A	-	-	-	+++
46	Sek34	-	-	-	-
47	Sek34b	-	-	-	-
48	Sje059-B	-	-	-	-
49	Sporogen AO-1	-	-	-	-
50	Synerazol	-	+	-	+++
51	Ta39s5	-	-	-	-
52	Tetrangulol	-	-	-	-
53	Watasemycin A	-	-	-	-
54	291-46	-	-	-	-

+, 30% inhibition; ++, 50% inhibition; +++, 80% inhibition; -, no inhibition; ±, increasing activity; NA not applicable. All experiments were performed in triplicate.

## Data Availability

Not applicable.

## Supplementary Materials

The following supporting information can be downloaded at: https://www.mdpi.com/article/10.3390/metabo12030246/s1.
