# Supplementary material for: Secondary Metabolites of Actinomycetales as Potent Quorum Sensing Inhibitors Targeting Gram-Positive Pathogens: In Vitro and In Silico Study
Source: Metabolites. 2022 Mar 15;12(3):246. doi: 10.3390/metabo12030246 (PMC8955454; doi:10.3390/metabo12030246)
Supplement: Supplementary file 1 [file metabolites-12-00246-s001.zip › metabolites-1587035-supplementary.pdf]

Supporting information

Secondary metabolites of actinomycetales as potent quorum sensing inhibitors targeting Gram-positive pathogens; *in vitro* and *in silico* study

Said E. Desouky [1, 2*], Mohammed A Abu-Elghait [2], Eman A. Fayed [3], Samy Selim [4], Basit Yousuf [1], Yasuhiro Igarashi [5], Basel A. Abdel-Wahab [6,7], Amnah Mohammed Alsuhaibani [8], Kenji Sonomoto [1], Jiro Nakayama [1]

Table S1. Structures of the selected compounds.

Fistupyrone	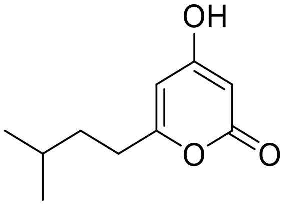
Lydicamycin	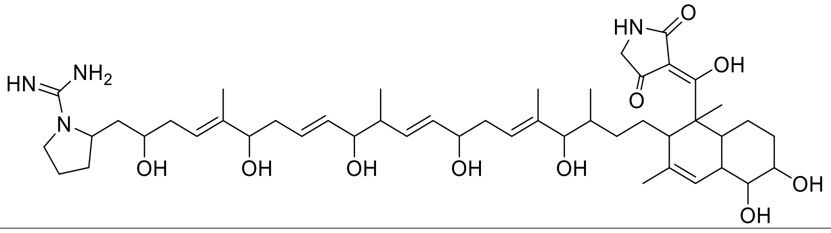
Watasemycin A	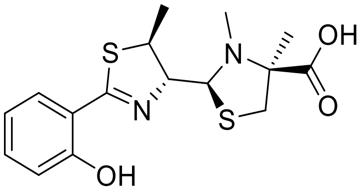
Rebeccamycin	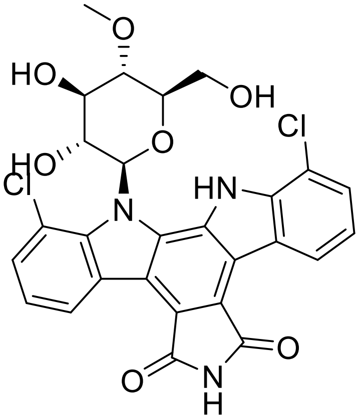
Nocardimicin H	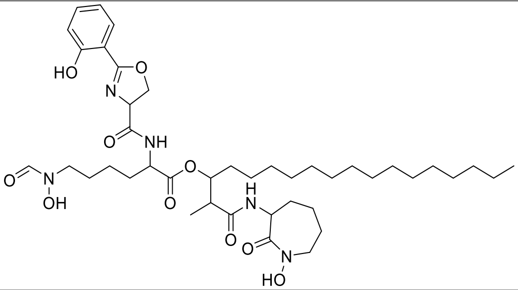
Rishirilide A	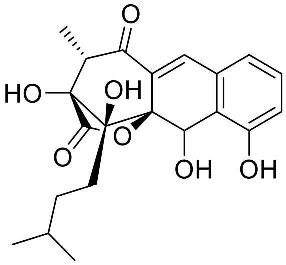

Lupinacidin C	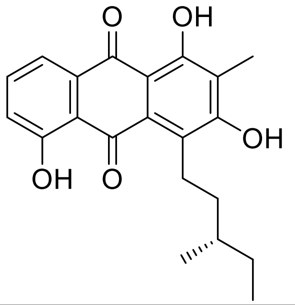
Rakicidin A	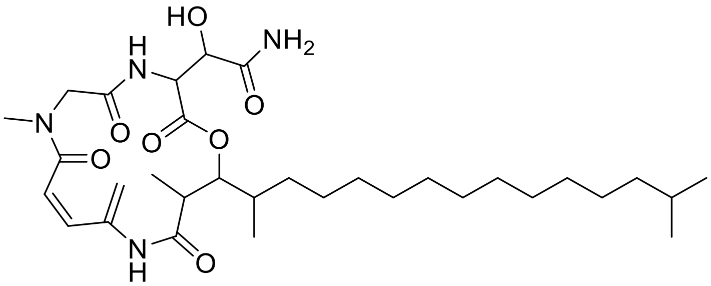
Rakicidin B	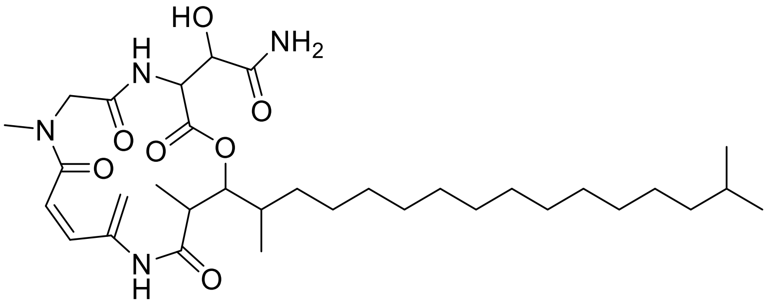
Phenalinolactone	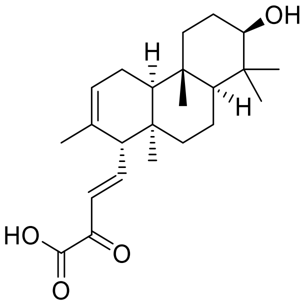
Myxochelinamide	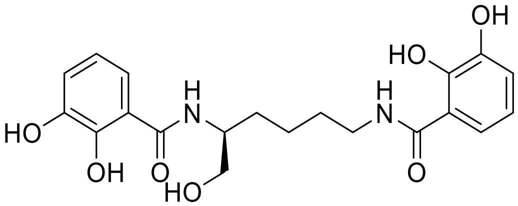

Pristinamycin IIA	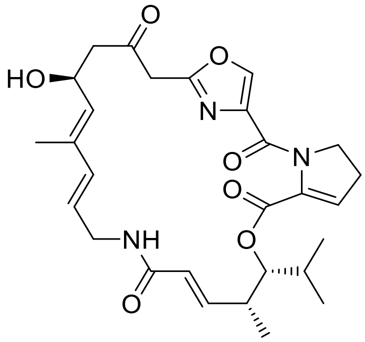
Pyridoxatin	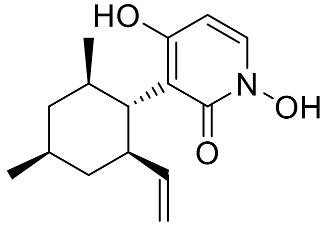
291-46	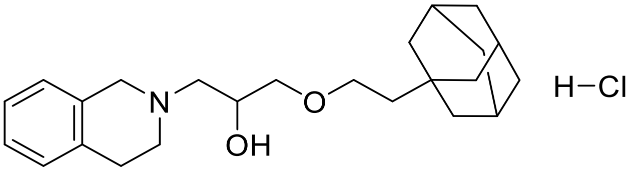
Preussin	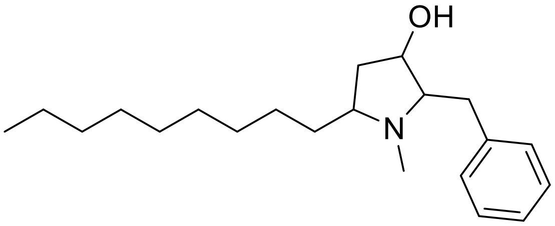
CHI15B (Tetrangulol)	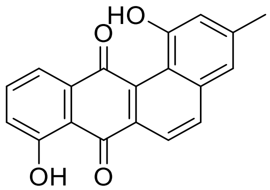
BU4664LAc	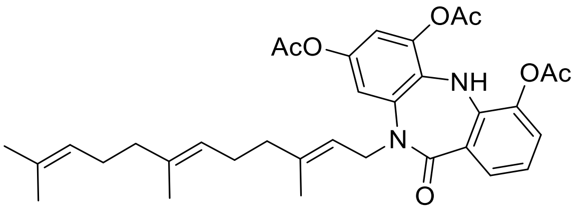
BU4664lMe	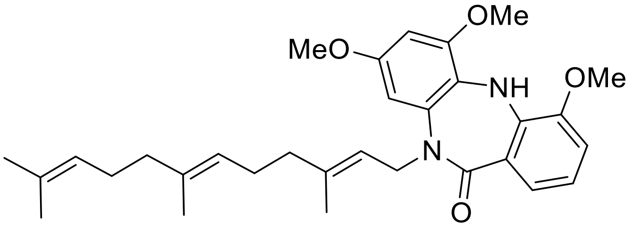

BU4664L	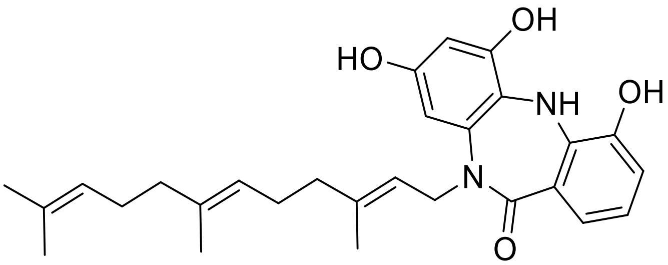
HF599 (maleimide)	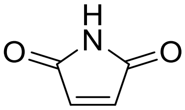
Abyssomicin I	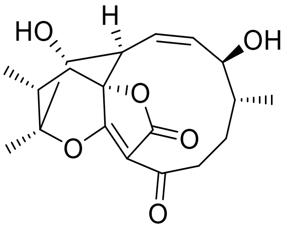
Sporogen AO-1	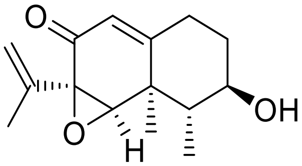
Mycolic acid	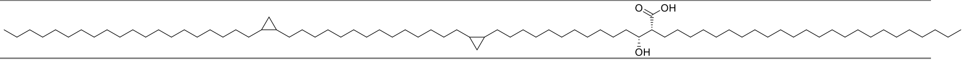
Chromone	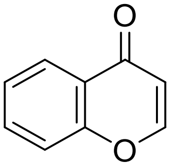
mucidone	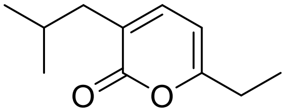
Furanone	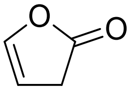
Maklamicin	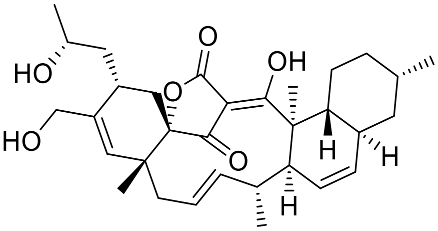
Coumarine	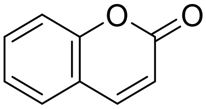

Collismycin	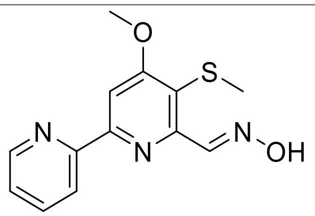
Borrelidin	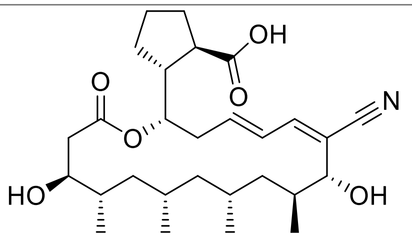
Radiciccol	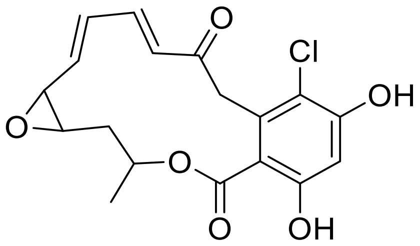
Decatromicin A	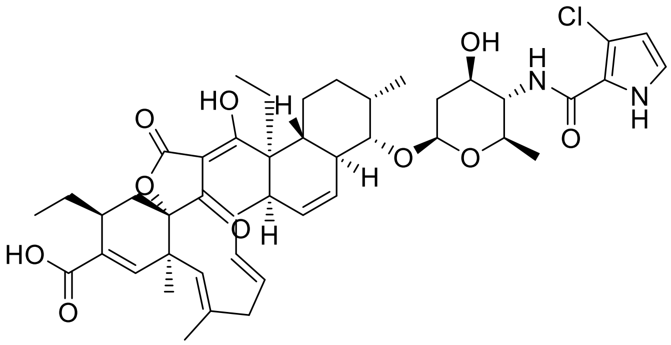
Decatromicin B	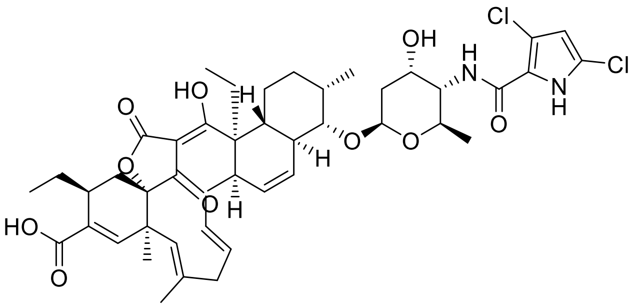
SEK34	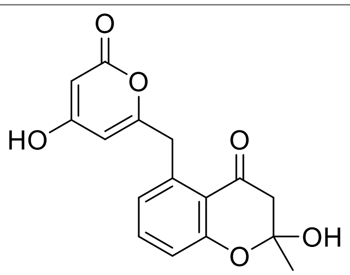

SEK34B	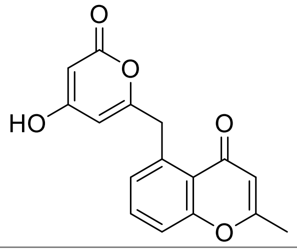
Lysolipin I	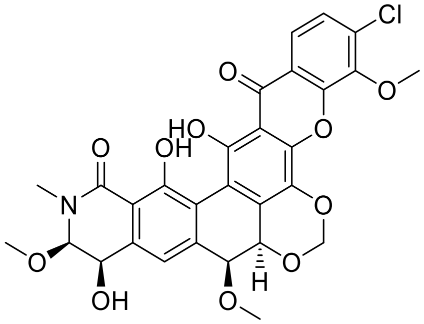
Geldanamycin	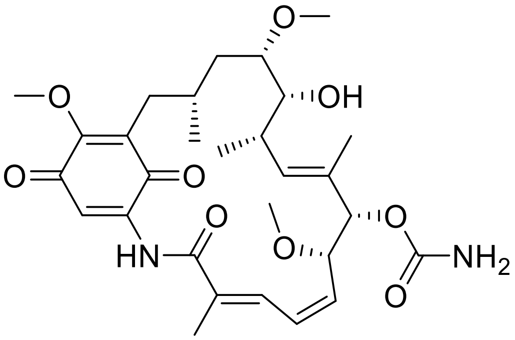
Enterocin	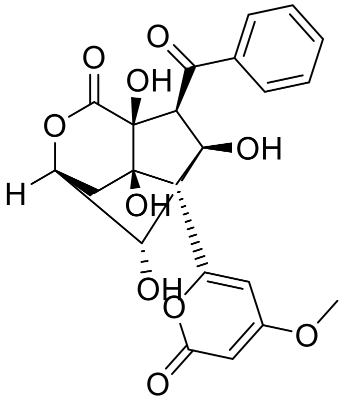
Questinomycin A	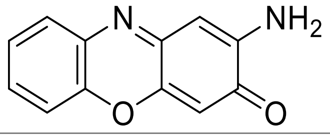

Pradimicin S	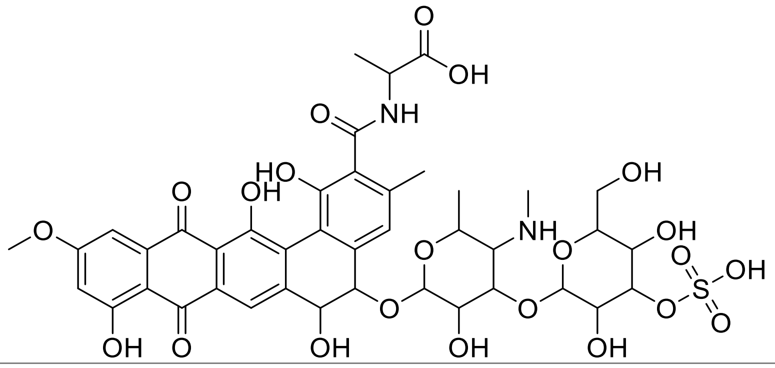
Synerazol	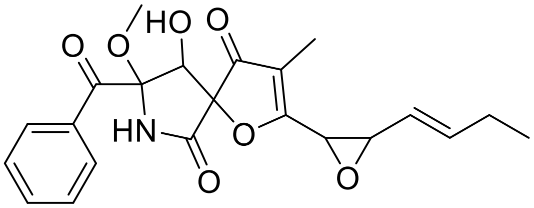
Pseurotin A	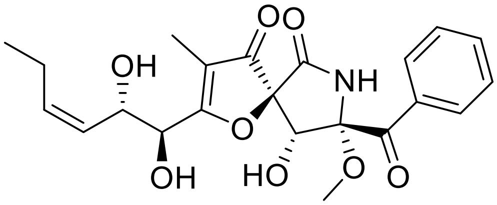
Okilactomycin	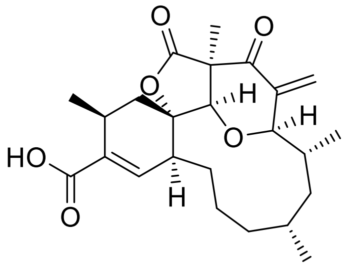
Leptomycin A	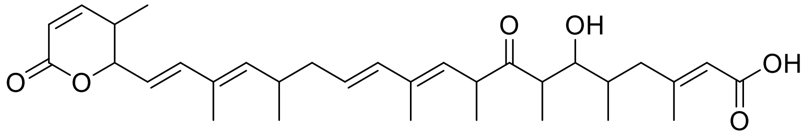
